# Cross-sectional associations of proteomic age acceleration with self-reported physical and mental health and depression symptoms among those with and without cancer

**DOI:** 10.1007/s11764-025-01803-7

**Published:** 2025-04-30

**Authors:** Matia Solomon, R Bhatia, S Wang, AH Blaes, EA Platz, W Guan, PI Jewett, A Prizment

**Affiliations:** 1 School of Medicine, University of Minnesota, Minneapolis, MN USA; 2 University of Michigan, Ann Arbor, MI USA; 3 Department of Laboratory Medicine and Pathology, School of Medicine, University of Minnesota, Minneapolis, MN USA; 4 Department of Epidemiology, Johns Hopkins Bloomberg School of Public Health, and the Sidney Kimmel Comprehensive Cancer Center at Johns Hopkins, Baltimore, MD USA; 5 Division of Biostatistics & Health Data Science, School of Public Health, University of Minnesota, Minneapolis, MN USA; 6 Division of Environmental Health Sciences, School of Public Health, University of Minnesota, Minneapolis, MN USA

**Keywords:** Age acceleration, Proteomic age, Cancer survivor, Cross-sectional, Quality of life, Depression

## Abstract

**Purpose:**

Accelerated aging may affect quality of life (QOL), and having cancer may further complicate that relationship. We assessed the cross-sectional association of a novel proteomic aging clock (PAC) with self-reported physical and mental health and depression symptoms among individuals with and without cancer.

**Methods:**

Data for this cross-sectional analysis came from the prospective Atherosclerosis Risk in Communities (ARIC) Study. We estimated proteomic age acceleration (PAA), i.e., the deviation of PAC from chronological age, at two ARIC visits (1990–1992 and 2011–2013). We used linear regression models to estimate the association of PAA and change in PAA with self-reported physical (PCS) and mental health (MCS) as well as depression symptoms, separately for cancer survivors and individuals without cancer.

**Results:**

Among cancer survivors, mean PCS, MCS, and depression scores were 44.0, 54.8, and 6.7, respectively; and 46.6, 55.3, and 6.5 among those without a cancer history. PAA was associated with lower PCS (adjusted coefficients per additional 5 years; cancer survivors, − 1.73, 95% CI − 3.11, − 0.35; without a cancer history, − 2.70, 95% CI − 3.90, − 1.51) and with higher depression scores among those without a cancer history (0.57, 95% CI 0.27, 0.88). Neither PAA nor change in PAA was associated with MCS in either population.

**Conclusion:**

We found some evidence for a cross-sectional association of PAA with self-reported physical health, but little evidence for an association with mental health and depression scores.

**Implications for Cancer Survivors:**

PAA may have stronger implications for physical than for mental health outcomes among those with and without cancer.

**Supplementary Information:**

The online version contains supplementary material available at https://doi.org/10.1007/s11764-025-01803-7.

## Introduction

There were an estimated 18.1 million cancer survivors in the USA as of January 2022 [1]. More than 60% of these individuals were aged 65 years or older, and their number is expected to grow to more than 70% by 2040. Cancer survivors are at risk of accelerated aging, a phenomenon in which one’s biological age, the accumulation of physiological changes over time, appears greater than one’s chronological age [2–5]. Cancer progression and treatments can contribute to accelerated aging through various mechanisms, such as genomic instability, telomere attrition, epigenetic alterations, loss of proteostasis, deregulated nutrient-sensing, mitochondrial dysfunction, cellular senescence, stem cell exhaustion, and altered intercellular communication [6]. Symptoms of accelerated aging in cancer survivors include frailty, cognitive impairment, organ dysfunction, secondary malignancies, and premature mortality [5].

To estimate biological age, various “aging clocks” have been devised using biomarkers including epigenomics, transcriptomics, and metabolomics [7–10]. Recently, it was shown that over 1000 plasma proteins have been found to systematically vary with age, and that proteomics can also be used to estimate biological age [11]. Some studies [12, 13], including ours [14], showed that proteomic age clocks (PACs) that are based on circulating protein biomarkers are strongly correlated with chronological age (*r* > 0.70), as a surrogate of biological age. The deviation of PAC from chronological age, coined PAA (or proteomic age acceleration), has been shown to be associated with age-related conditions, such as all-cause mortality, frailty, cancer, and dementia in the general population [13, 15, 16].

Accelerated aging likely has consequences for quality of life (QOL) [17, 18]. For example, older adults put emphasis on functional ability, living without significant impairment, participation in leisurely activities, and relationships with friends and family [19–22]. Some studies suggest that older adults self-report better mental health, including less depression, but worse physical quality of life compared to younger adults [23–30], and these associations are potentially modified by having had cancer. However, individuals of the same chronological age may have different biological age, and while numerous studies have assessed the relationship between chronological age and QOL [18, 22, 23, 26, 27, 30], little is known about the association between accelerated aging and QOL separately in cancer survivors and those without a cancer history. Additionally, it is plausible that speed of accelerated aging matters [31]: changes in the speed of accelerated aging over time could have potential consequences for QOL. For example, it is possible that having cancer is related with the speed at which one’s biological age changes [5]. Further, a plausible hypothesis is that the speed of change of biological age may interact with other effects of cancer and uniquely impact QOL experiences, which would mean that it would be important to capture change in accelerated age in addition to accelerated age itself.

Based on the above described recently growing interest in and research on biological age that may differ from chronological age, but dearth of previous studies that have linked biological age to QOL outcomes, we conducted a study to explore the cross-sectional association between a novel PAC [14], developed and validated by our study team, with self-reported physical and mental health as well as depression symptoms among individuals with and without cancer. Our aim was to identify potential links of QOL with biological age based on data from the Atherosclerosis Risk in Communities (ARIC). We hypothesized that PAA would be associated with lower physical and mental QOL and more depressive symptoms in both cancer survivors and those without a history of cancer. Further, we expected this association to be stronger among cancer survivors compared to those without a history of cancer due to potential other factors associated with cancer, such as past cancer treatment.

## Methods

### Data source and study design

Data for this study came from the Atherosclerosis Risk in Communities (ARIC). ARIC (RRID: SCR_021769) is an ongoing prospective population-based cohort study. We used data from ARIC visits 2 (1990–1992) and 5 (2011–2013). Although we used data from these two ARIC visits, our study design was cross-sectional by comparing outcome measures captured at visit 5 cross-sectionally with (1) proteomic age at visit 5 and (2) change in proteomic age from visit 2 until visit 5.

## Participants and sampling

A total of 15,792 mostly White and Black men and women aged 45–64 years were enrolled between 1987 and 1989, with 10 follow-up visits to date. Incident cancer has been ascertained in 4613 individuals (recorded between 1987 and 2015 using cancer registry data and medical records).

Of 6544 participants at visit 5, 6526 identified as Black or white (Supplemental Fig. 1). We stratified by race, excluding individuals who were not White or Black given the small sample size of that group. We excluded 313 individuals with prevalent cancer at visit 1 because their time since diagnosis was unknown and another 73 because their cancer status at enrollment was unknown. We further limited participants with cancer at visit 5 to those whose diagnosis dated to at least a year prior to their visit 5, excluding another 54 individuals, in order to minimize the effect of active cancer treatment on protein concentrations. Of the remaining, 1243 did not have protein measures, and another 29 did not report any outcomes at visit 5 (physical and mental health QOL score, depression score). Lastly, we excluded 2451 individuals who had been included in the training dataset to develop the PAC, bringing the final analytical sample size to *N* = 2363. Among those, 866 participants were cancer survivors at visit 5.

## Measurements and outcomes

Our main exposure was the proteomic age at visit 5 and change in proteomic age from visit 2 to visit 5 (mean time interval 20.8 ± 1.0 years). Details of the development of the PACs at visit 2 and visit 5 were developed and are described elsewhere [14]. Briefly, PACs were trained against chronological age at visit 2 and visit 5 in two-thirds of randomly selected ARIC participants who did not have cancer or other chronic diseases at the corresponding visit using elastic net regression (alpha = 0.5). These PACs were internally validated in the remaining cancer-free participants at the corresponding visit. Subsequently, these PACs were used to estimate participants’ PAA at ARIC visits 2 and 5 (two visits when the required protein measurements were available) of individuals with malignancies (hematologic or solid and individuals without any malignancies). Of note, we included only one-third of participants without malignancies at visit 2 and visit 5, since two-thirds of disease-free participants were included in the training set that was used to develop the cancer-free PAC at the corresponding visit. PAA was then calculated at visit 2 and visit 5 as the residuals from regressing PAC at the corresponding visit on chronological age; and the increase/decrease in PAA was measured as PAA at visit 5 minus PAA at visit 2.

Our primary outcomes were physical and mental health and depression reported at ARIC visit 5. The ARIC study administered the 12-Item Short Form Health Survey (SF- 12) Version 2 to assess health-related QOL [32]. The 12 items cover physical functioning, physical roles, bodily pain, general health, energy/fatigue, social functioning, socio-emotional roles, and mental health. The overall SF- 12 score comprises a Physical Component Score (PCS) and a Mental Component Score (MCS) [32]. The norm-based SF- 12 subscores have a mean of 50 and a standard deviation of 10 in the general population; the score ranges in our study population were 4.6–66.6 for the PCS, and 19.4–73.6 for the MCS. The ARIC study administered the Center for Epidemiological Studies Depression Scale (CES-D) to assess depression [33]. CES-D scores can range between 0 and 22, and any score above 9 indicates significant depressive symptoms.

## Statistical analysis and covariates

We ran descriptive statistics (means, standard deviation, frequencies) of our study population (demographic and clinical characteristics, outcomes, and PAC). Separately in cancer survivors and those without a cancer history, we used linear regression analyses, with PAA at visit 5 and change in PAA between visit 2 and visit 5 as the main predictors, and the SF- 12 PCS and MCS as well as the CES-D depression scores at visit 5 as outcomes. We ran our models both unadjusted and adjusted for the covariates listed above. We ran additional models for survivors of specific cancer types with the largest number of cases: breast cancer, prostate cancer, and colorectal cancer. These models were additionally adjusted for cancer stage at diagnosis. Analyses were conducted using SAS 9.4. All tests were two-sided with alpha = 0.05 and *P*-values < 0.05 were considered significant.

Covariates for our linear regression models were identified a priori. We used sociodemographic factors collected at ARIC visit 1: race (White, Black), gender (measured at the time of visit 1 as female, male), partner status (with/without partner), income (< $25,000, $25,000–49,999, $50,000–99,999, ≥ $100,000), education (less than high school, high school or vocational school, and at least some college), and study center (Forsyth County, NC; Jackson, MS; Minneapolis, MN; Washington County, MD). We used factors that are potential confounders or may affect plasma protein levels or that are upstream of accelerated aging that were collected at visit 5: kidney function (i.e., estimated glomerular filtration rate [eGFR] in mL/min/1.73 m^2^, continuous), body mass index (BMI, continuous), diabetes (yes/no), cardiovascular disease (yes/no), physical activity (a continuous indicator of intensity of physical activity) [34], and smoking status (current smoker, former smoker, never smoker, unknown). In the model with change in PAA from visit 2 to visit 5 as the exposure, we also adjusted for PAA at visit 2, including in the otherwise unadjusted model. Additionally, in the analysis among those with cancer, we adjusted for time since cancer diagnosis (continuous), and an indicator of whether the cancer diagnosis had occurred between ARIC visits 2 and 5.

## Results

### Characteristics of the study population

Of the 2363 study participants, 866 were cancer survivors (32.2% prostate, 21.8% breast) at visit 5, while 1497 did not have a cancer history (Table 1). The mean age of participants at visit 5 was 75.8 ± 5.2 years. About half (54.5%) of participants were female (46.3% among those with cancer, 58.3% among those without cancer, *P* < 0.0001); 80.7% identified as White; 65.3% reported having a partner; 27.4% reported incomes lower than $25,000; 14.1% reported not having completed high school and 43.9% reported at least some college. Those with cancer, compared to those without, more often reported having a partner (68.7% versus 63.4%), were slightly older (mean age 76.4 ± 5.2 years versus 75.4 ± 5.1 years), and more often reported having prevalent CVD (28.5% versus 24.1%) and lower eGFR (mean 65.5 ± 19.6 years versus 68.7 ± 18.8 mL/min/1.73 m^2^).

**Table 1:** Characteristics of visit 5 study population, *N*=2363, Atherosclerosis Risk in Communities Study

Participant characteristics at visit 5*	Everyone (*N*=2363)		Without cancer (*N*=1497)		With cancer (*N*=866)		*P*
Age, mean (SD)	75.8 (5.2)		75.5 (5.2)		76.4 (5.2)		**<0.0001**
Body mass index, mean (SD)	28.8 (5.7)		28.8 (5.8)		28.8 (5.7)		0.96
eGFR, mean (SD)	67.5 (19.1)		68.7 (18.8)		65.5 (19.6)		**0.0001**
Physical activity score, mean (SD)	2.6 (0.8)		2.6 (0.8)		2.5 (0.8)		**0.007**
	*N*	**%**	*N*	**%**	*N*	**%**	
Gender							**<0.0001**
Female	1,140	54.5	876	58.5	401	46.3	
Male	951	45.5	621	41.5	465	53.7	
Race							0.88
Black	404	19.3	289	19.3	165	19.1	
White	1687	80.7	1208	80.7	701	81	
Partner status at visit 5							**0.01**
Not partnered	698	34.7	531	36.6	258	31.3	
Partnered	1,316	65.3	919	63.4	566	68.7	
Income at visit 5							0.07
< $25,000	527	27.4	387	28.2	201	25.3	
$25,000–49,999	650	33.8	455	33.1	285	35.8	
$50,000–99,999	555	28.9	408	29.7	218	27.4	
≥$100,000	191	9.9	123	9.0	92	11.6	
Education level at visit 5							0.15
Less than completed high school	294	14.1	206	13.8	119	13.7	
High school or vocational school	880	42.1	662	44.3	350	40.4	
At least some college	917	43.9	627	42.0	397	45.8	
Smoking status at visit 5							0.29
Current smoker	126	5.5	80	5.5	46	5.4	
Former smoker	1113	49.1	695	47.7	438	51.5	
Never smoker	865	37.5	567	38.9	298	35.0	
Unknown	185	8.0	116	8.0	69	8.1	
CVD status at visit 5							**0.02**
No	1725	74.3	1114	75.9	611	71.5	
Yes	597	25.7	353	24.1	244	28.5	
Diabetes status at visit 5							0.08
No	1526	66.3	988	67.6	538	64.1	
Yes	775	33.7	473	32.4	302	36.0	
Cancer type at visit 5							
Hematologic					47	5.4	
Brain					14	1.6	
Breast					181	20.9	
Colorectal					83	9.6	
Gynecologic					59	6.8	
Head/neck					7	0.8	
Melanoma					42	4.8	
Other digestive					18	2.1	
Prostate					267	30.8	
Respiratory system					30	3.5	
Urinary tract					81	9.4﻿	
Other					37	4.3	

*At visit 5 unless noted otherwise

## Outcome and exposure distributions

**Fig. 1:**
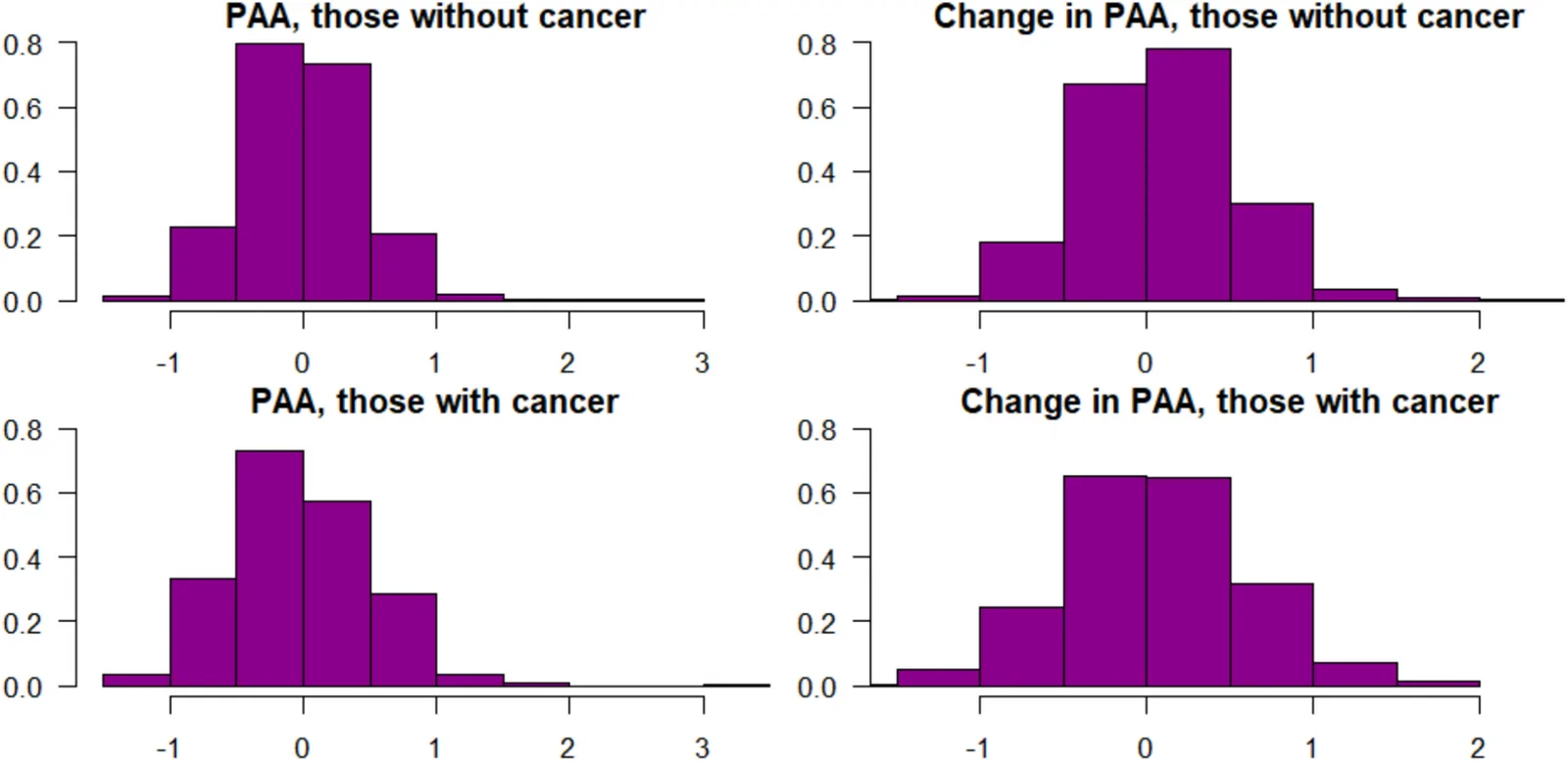
Distribution of proteomic age at visit 5 and change in proteomic age between visits 2 and 5. *N* = 2363, Atherosclerosis Risk in Communities Study

Mean PCS (physical QOL) scores were significantly lower (*P* < 0.0001) among those with cancer (44.0 ± 10.9 compared to 46.6 ± 10.3 among those without cancer). The mean MCS (mental QOL) score was 54.8 ± 8.3 among those with cancer (55.3 ± 7.7 among those without cancer), and the mean depression score was 6.7 ± 2.5 among those with cancer (6.5 ± 2.5 among those without cancer). Comparisons of the MCS and depression scores between these groups were not significant (*P* > 0.05). Proteomic age at visit 5 ranged from − 1.3 to 2.6 among those without cancer (− 1.4 to 3.0 among those with cancer), and change in proteomic age ranged from − 1.9 to 2.1 among those without cancer (− 1.6 to 1.7 among those with cancer, Fig. 1).

## Associations of PAA with outcomes

Associations of PAA and change in PAA with PCS, MCS, and depression scores are shown in Table 2. Interaction terms between cancer status and the main exposures were not significant for any of the outcomes. Nevertheless, we stratified our analyses by cancer status. Among cancer survivors, PAA was associated with lower PCS scores (per additional 5 years of PAA, adjusted coefficient − 1.73, 95% CI − 3.11, − 0.35). Change in PAA was also associated with PCS scores in the unadjusted model (per additional 5 years of change in PAA, unadjusted coefficient − 3.20, 95% CI − 4.80, − 1.61).

**Table 2:** Associations of age acceleration at visit 5 and change (between visit 2 and visit 5) in age acceleration with visit 5 physical and mental quality of life and depression scores, by cancer status (with cancer, *N*=1497; without cancer *N*=866), Atherosclerosis Risk in Communities Study

Outcome	Predictor		Without cancer (*N*=1497)		With cancer (*N*=866)	
			Coefficient (95% CI)	*P*	Coefficient (95% CI)	*P*
Physical health score	Age acceleration, per 5 years	Unadjusted*	**−2.94 (−4.15,−1.72)**	**<0.0001**	**−3.16 (−4.54,−1.77)**	**<0.0001**
		Adjusted**	**−2.27 (−3.53,−1.01)**	**0.004**	**−2.11 (−3.59,−0.64)**	**0.005**
		Adjusted, more covariates***	**−2.70 (−3.90,−1.51)**	**<0.0001**	**−1.73 (−3.11,−0.35)**	**0.01**
	Change in age acceleration, per 5 years	Unadjusted*	**−3.08 (−4.54,−1.62)**	**<0.0001**	**−3.20 (−4.80,−1.61)**	**<0.0001**
		Adjusted**	**−2.32 (−3.81,−0.82)**	**0.002**	**−2.20 (−3.88,−0.53**	**0.001**
		Adjusted, more covariates***	**−3.02 (−4.43,−1.60)**	**<0.0001**	−1.53 (−3.09,0.04)	0.06
Mental health score	Age acceleration, per 5 years	Unadjusted*	−0.71 (−1.61,0.20)	0.12	−0.31 (−1.38,0.76)	0.57
		Adjusted**	−0.45 (−1.42,0.51)	0.36	−0.42 (−1.59,0.75)	0.49
		Adjusted, more covariates***	−0.10 (−1.11,0.92)	0.85	−0.45 (−1.63,0.73)	0.46
	Change in age acceleration, per 5 years	Unadjusted*	−0.36 (−1.46,0.74)	0.52	−0.86 (−2.08,0.36)	0.17
		Adjusted**	−0.20 (−1.36,0.97)	0.74	−0.92 (−2.23,0.39)	0.17
		Adjusted, more covariates***	0.10 (−1.11,1.32)	0.87	−0.97 (−2.28,0.35)	0.15
Depression score	Age acceleration, per 5 years	Unadjusted*	**0.48 (0.19,0.77)**	**0.001**	0.31 (−0.02,0.64)	0.06
		Adjusted**	**0.50 (0.20,0.79)**	**0.001**	0.16 (−0.19,0.51)	0.36
		Adjusted, more covariates***	**0.57 (0.27,0.88)**	**0.0003**	0.24 (−0.11,0.59)	0.17
	Change in age acceleration, per 5 years	Unadjusted*	**0.51 (0.16,0.86)**	**0.004**	0.37 (−0.01,0.75)	0.05
		Adjusted**	**0.57 (0.22,0.92)**	**0.001**	0.21 (−0.18,0.61)	0.30
		Adjusted, more covariates***	**0.67 (0.31,1.03)**	**0.0003**	0.28 (−0.11,0.68)	0.16

*The models with change in age acceleration between visit 2 and visit 5 as the predictor were adjusted for age acceleration at visit 2

**Covariates: Race, gender, partner status income, education, partner status, EGFR, time since diagnosis, cancer stage, and an indicator whether the cancer diagnosis occurred between ARIC visit 2 and visit 5, BMI at visit 5, smoking, physical activity, and indicators whether individuals had diabetes or CVD

Neither PAA nor change in PAA between visits 2 and 5 was associated with higher depression scores among cancer survivors. Among those without a cancer history, PAA was associated with lower PCS scores (per additional 5 years of PAA, adjusted coefficient, − 2.70, 95% CI − 3.90, − 1.51), as was change in PAA (per additional 5 years of change in PAA, adjusted coefficient − 3.02, 95% CI − 4.43, − 1.60). PAA and change in PAA were also associated with higher depression scores (per additional 5 years of PAA, adjusted coefficient 0.57, 95% CI 0.27, 0.88). In neither cancer survivors nor those without a cancer history was PAA or change in PAA associated with MCS scores (Table 2).

When stratifying by most common cancer sites (breast, colorectal, prostate cancer; Table 3), in the unadjusted models, PAA was associated with lower PCS scores among those with breast cancer (per additional 5 years of PAA, unadjusted coefficient − 4.01, 95% CI − 7.09, − 0.93) and among those with prostate cancer in the unadjusted model (per additional 5 years of PAA, unadjusted coefficient − 2.58, 95% CI − 4.92, − 0.24). In these models, we additionally tested for interaction between stage and the main predictors. We found no significant interaction except between PAA and stage among those with prostate cancer and with depression as outcome (*P* = 0.04), and between change in PAA and stage among those with breast cancer with PCS scores as outcomes (*P* = 0.04).

**Table 3:** Unadjusted and adjusted associations of proteomic age acceleration at visit 5 and change (between visit 2 and visit 5) in age acceleration with physical and mental quality of life and depression scores, by cancer site (breast cancer, colorectal cancer, and prostate cancer), Atherosclerosis Risk in Communities Study

Outcome	Predictor	Breast cancer (*N* = 181)		Colorectal cancer (*N* = 83)		Prostate cancer (*N* = 267)	
		Coefficient (95% CI)	*P*	Coefficient (95% CI)	*P*	Coefficient (95% CI)	*P*
Unadjusted model*							
Physical health score	Age acceleration, per 5 years	** − 4.01 (− 7.09, − 0.93)**	**0.01**	− 2.96 (− 7.41,1.49)	0.19	** − 2.58 (− 4.92, − 0.24)**	**0.03**
	Change in age acceleration, per 5 years	− 3.42 (− 6.98,0.15)	0.06	− 3.34 (− 8.18,1.49)	0.18	** − 3.04 (− 5.75, − 0.32)**	**0.03**
Mental health score	Age acceleration, per 5 years	0.6 (− 1.7,2.9)	0.61	2.63 (− 1.43,6.68)	0.20	− 1.01 (− 3.04,1.03)	0.33
	Change in age acceleration, per 5 years	− 0.17 (− 2.83,2.48)	0.90	2.22 (− 2.14,6.59)	0.32	− 1.46 (− 3.74,0.83)	0.21
Depression score	Age acceleration, per 5 years	0.59 (− 0.04,1.23)	0.07	− 1.15 (− 2.54,0.24)	0.10	0.3 (− 0.27,0.87)	0.31
	Change in age acceleration, per 5 years	**0.78 (0.03,1.52)**	**0.04**	− 1.14 (− 2.63,0.35)	0.13	0.34 (− 0.29,0.97)	0.29
Adjusted model**							
Physical health score	Age acceleration, per 5 years	− 1.04 (− 4.74,2.65)	0.58	N/A		− 0.34 (− 2.7,2.02)	0.78
	Change in age acceleration, per 5 years	− 0.47 (− 4.82,3.87)	0.83			− 0.65 (− 3.43,2.14)	0.65
Mental health score	Age acceleration, per 5 years	− 1.01 (− 4.18,2.16)	0.53			− 1.04 (− 3.25,1.17)	0.36
	Change in age acceleration, per 5 years	− 0.02 (− 3.57,3.54)	0.99			− 0.85 (− 3.34,1.63)	0.50
Depression score	Age acceleration, per 5 years	1 (0.11,1.89)	0.03			− 0.07 (− 0.68,0.53)	0.81
	Change in age acceleration, per 5 years**	0.94 (− 0.07,1.94)	0.07			− 0.17 (− 0.86,0.52)	0.63

*The models with change in age acceleration between visit 2 and visit 5 as the predictor were adjusted for age acceleration at visit 2

**Covariates: Race, gender, partner status income, education, partner status, EGFR, time since diagnosis, cancer stage, and an indicator whether the cancer diagnosis occurred between ARIC visit 2 and visit 5, BMI at visit 5, smoking, physical activity, and indicators whether individuals had diabetes or CVD

## Sensitivity analyses

We ran several sensitivity analyses (Supplemental Tables 1, 2, 3). First, we re-ran models among those without cancer excluding 272 individuals who developed cancer after visit 5 (Supplemental Table 1). These individuals might have already had cancer at visit 5 despite not yet having been diagnosed. Second, we re-ran models among cancer survivors excluding participants who were cancer-free at visit 1, but developed cancer before visit 2, i.e., before the first measurement of PAA in this study (Supplemental Table 2). We also added a set of models additionally adjusted for chronological age at visit 5 to the main models. (Supplemental Table 3). We additionally tested for interaction of PAA and change in PAA with time since diagnosis. None of the interaction terms was significant (data not shown).

Lastly, we re-ran the fully adjusted model among cancer survivors in which we excluded covariates that had been part of the adjusted model only for cancer survivors (time since diagnosis and an indicator of whether the cancer diagnosis occurred between ARIC visits 2 and 5, Supplemental Table 4). The results from these sensitivity models were not meaningfully different from the corresponding main models.

## Discussion

In this cross-sectional study, we aimed to identify the associations of proteomic age (PAA) and change in PAA with QOL in those with and without cancer since biological age may be more relevant to QOL than chronological age. We found that PAA at visit 5 (2011–2013, aged 66–90 years) and positive change in PAA between visits 2 (1990–1992, aged 46–70 years) and 5 were associated with lower PCS scores among cancer survivors and individuals without a cancer history, supporting our hypotheses. We also found that PAA and increases in PAA were associated with higher depression scores among those without cancer, but not among those with cancer, and not with mental health scores in either population, contradicting our hypotheses. We found no evidence for interaction of PAA or change in PAA with cancer status and effect sizes were similar in cancer survivors and those without a cancer history.

Our finding that mean physical QOL scores were lower in those among cancer is consistent with prior work [35–38]. Further, our findings suggest that elevated PAA is associated with lower physical QOL, without evidence for interaction, among those with and without cancer, suggesting that proteomic age acceleration, regardless of health status, increases risk for impairments in physical well-being. This observation parallels known associations of chronological age with reduced physical function [39, 40]. When stratifying the unadjusted model by the most common cancer types (breast, colorectal, and prostate cancer), we found some nuances in the effect sizes, but overall similar findings as in the non-stratified main analysis, suggesting that associations did not differ by cancer type, similar to the lack of evidence for interaction by cancer status in our study.

We provided estimates for outcome differences per additional 5 years in PAA and per 5-year increase in change of PAA (i.e., increasing speed of aging). In our study population, the Q1 to Q3 value distributions for PAA and change in PAA ranged from approximately − 1.6 to + 1.5 years, and from − 1.3 to + 1.9 years, respectively. Therefore, our coefficients per 5 years represent outcome changes among individuals with greater-than-average PAA and change in PAA. The differences in outcome scores (coefficient effect sizes approximately 1.5–3 for PCS, < 1 for MCS, 0.2–0.4 for depression) associated with these above-average 5-year differences in PAA and change in PAA are small compared with the overall outcome scores in our population (approximately 44–47 for PCS, 55 for MCS, and 6.5 for depression). Hence, the estimated effect sizes of the observed associations are small. For most individuals, the outcome effect sizes will likely be smaller because their PAA and change in PAA are lower.

Neither PAA nor increases in PAA were associated with differences in mental health QOL scores in either cancer survivors or those without a cancer history, and they were associated with differences in depression scores among those without cancer only. Further, overall MCS and depression scores did not differ significantly between those with and without cancer. These findings are perhaps counterintuitive and contradict prior evidence of mental health sequelae of cancer [38, 41]. While we have no immediate interpretation of those findings, it is possible that self-reported mental health varies less between those with and without cancer because older adults tend to generally self-report better mental health [23–30]. It is also possible that these findings may reflect that these are cancer survivors. Participants had to still be alive and well enough to respond to the questionnaires and attend the study visit which may have introduced survival bias and potentially elevated mental health and low depression outcomes.

Additionally, while lasting cancer sequelae may impact QOL among many of those who have had cancer [38, 42], some long-term cancer survivors return to much improved general health long-term, and this group could have been overrepresented among the long-term cancer survivors in our study population. Further, many of the cancer survivors had prostate and breast cancer, and these cancer patients, if without metastases, often return to their pre-cancer health status [43–47].

We found no evidence with regard to any of our three primary outcomes that associations of PAA and change in PAA were stronger among those with cancer compared to those without cancer, contradicting our original hypothesis: we had expected that the associations would be stronger among those with cancer. This is consistent with our finding that interaction terms by cancer status were not significant; and estimated effect sizes were similar among cancer survivors and those without a cancer history. It is possible that our findings were impacted by survival bias since most individuals with cancer were long-term cancer survivors, many of whom were potentially not on current treatment. More than half (54%) of the cancer survivors in our study were diagnosed with breast and prostate cancers. When breast or prostate cancer is diagnosed at localized stages, it is often treated with surgery and radiation therapy, and for breast cancer, with maintenance therapy (e.g., tamoxifen) for several years, after which many patients eventually return to their normal lives. Future studies should assess whether findings differ in cohorts of individuals recently diagnosed with cancer.

Limitations of our study included the cross-sectional nature of our data. The only clinical cancer characteristics consistently available for all cancer survivors in our study were cancer type and time since diagnosis; for example, we had no information on treatments received. Cancer stage was available only among those with the most common cancers (breast, prostate, colorectal). We added cancer stage as a covariate in our sensitivity analysis stratified by these three cancer sites. Despite adjusting for time since diagnosis, we did not capture participants’ specific survivorship phase (e.g., initial treatment, long-term maintenance treatment, long-term survival post-treatment) which could be a potential effect modifier. The study population also represents an older population of cancer survivors. Our findings are not generalizable to young adult cancer survivors among whom PAA may have different impacts on QOL outcomes. We excluded ARIC participants who had been in the training dataset to develop the PAA. Since ARIC participants had to be disease-free to be in the training dataset, this may have biased our remaining cancer-free dataset to represent those who were generally less healthy.

## Conclusion

In our study, we linked PAA and change in PAA to QOL outcomes among those with and without cancer. We provided partial support for our hypotheses: we found some evidence for an association, with small effect sizes, of PAA with physical QOL and depression, but no evidence for an association with mental health. We found no evidence for interaction of PAA or change in PAA with cancer status. Together, these findings suggest that PAA is more strongly associated with physical health outcomes than with mental health outcomes among both those with and without cancer. Future research should assess these associations among individuals recently diagnosed with cancers, as well as among individuals with specific cancers since sample sizes in our analyses stratified by cancer site were limited. Furthermore, longitudinal assessments are needed to investigate whether greater change in PAA is associated with faster declines in health-related QOL over time.

## Supplementary Information

Below is the link to the electronic supplementary material.

Supplementary file1 (DOCX 72.2 KB)

## Data Availability

No datasets were generated or analysed during the current study.
