# Supplementary material for: Cross-sectional associations of proteomic age acceleration with self-reported physical and mental health and depression symptoms among those with and without cancer
Source: J Cancer Surviv. 2025 Apr 30;20(Suppl 1):239–48. doi: 10.1007/s11764-025-01803-7 (PMC13676640; doi:10.1007/s11764-025-01803-7)
Supplement: Supplementary file 1 [file 11764_2025_1803_MOESM1_ESM.docx]

**Supplemental Figure 1** Flow chart: study population, Atherosclerosis Risk in Communities Study

**Supplemental Table 1** Sensitivity analysis: Associations of proteomic age acceleration and change (between visit 2 and visit 5) in age acceleration with visit 5 physical and mental quality of life and depression scores, excluding those who were classified as ‘without cancer’ in the main model, but who were diagnosed with cancer after visit 5. N=1,225, Atherosclerosis Risk in Communities Study

| **Outcome** | **Predictor** | **Unadjusted*** | | **Adjusted**** | | **Adjusted, more covariates***** | |
| --- | --- | --- | --- | --- | --- | --- | --- |
|  |  | **Coefficient**  **(95% CI)** | **P** | **Coefficient**  **(95% CI)** | **P** | **Coefficient**  **(95% CI)** | **P** |
| **Those without cancer (N=1,225)** | | | | | | | |
| Physical health score | Age acceleration, per 5 years | **-2.97 (-4.35,-1.59)** | **<.0001** | **-2.1 (-3.54,-0.66)** | **0.004** | **-2.66 (-4.02,-1.29)** | **0.0001** |
|  | Change in age acceleration, per 5 years** | **-3.00 (-4.66,-1.35)** | **0.0004** | **-2.66 (-4.02,-1.29)** | **0.0001** | **-2.64 (-4.25,-1.02)** | **0.001** |
| Mental health score | Age acceleration, per 5 years | -1 (-2.03,0.02) | 0.06 | -0.73 (-1.83,0.38) | 0.20 | -0.3 (-1.46,0.86) | 0.61 |
|  | Change in age acceleration, per 5 years** | -0.77 (-2.03,0.49) | 0.23 | -0.3 (-1.46,0.86) | 0.61 | -0.11 (-1.5,1.27) | 0.87 |
| Depression score | Age acceleration, per 5 years | **0.48 (0.16,0.81)** | **0.004** | **0.48 (0.14,0.82)** | **0.01** | **0.48 (0.12,0.83)** | **0.01** |
|  | Change in age acceleration, per 5 years** | **0.63 (0.23,1.02)** | **0.002** | **0.48 (0.12,0.83)** | **0.01** | **0.62 (0.21,1.04)** | **0.003** |

* The models with change in age acceleration between visit 2 and visit 5 as the predictor were adjusted for age acceleration at visit 2.

** Covariates: Race, gender, partner status income, education, partner status, EGFR, study site.

*** Additional covariates: BMI at visit 5, smoking, physical activity, and indicators whether individuals had diabetes or CVD

**Supplemental Table 2** Sensitivity analysis: Associations of proteomic age acceleration and change (between visit 2 and visit 5) in age acceleration with visit 5 physical and mental quality of life and depression scores, excluding those from the cancer survivor group who were diagnosed with cancer between visit 1 and visit 2. N=819, Atherosclerosis Risk in Communities Study

| **Outcome** | **Predictor** | **Unadjusted** | | **Adjusted*** | | **Adjusted, more covariates**** | |
| --- | --- | --- | --- | --- | --- | --- | --- |
|  |  | **Coefficient**  **(95% CI)** | **P** | **Coefficient**  **(95% CI)** | **P** | **Coefficient**  **(95% CI)** | **P** |
| **Those with cancer (N=819)** | | | | | | | |
| Physical health score | Age acceleration, per 5 years | **-3.2 (-4.63,-1.77)** | **<.0001** | **-2.23 (-3.75,-0.71)** | **0.004** | **-1.77 (-3.2,-0.34)** | **0.02** |
|  | Change in age acceleration, per 5 years** | **-3.34 (-4.99,-1.69)** | **<.0001** | **-2.33 (-4.07,-0.59)** | **0.009** | -1.53 (-3.16,0.11) | 0.07 |
| Mental health score | Age acceleration, per 5 years | -0.35 (-1.46,0.76) | 0.54 | -0.41 (-1.63,0.81) | 0.51 | -0.4 (-1.64,0.83) | 0.52 |
|  | Change in age acceleration, per 5 years** | -0.91 (-2.18,0.37) | 0.16 | -0.92 (-2.29,0.46) | 0.19 | -0.96 (-2.34,0.43) | 0.18 |
| Depression score | Age acceleration, per 5 years | 0.33 (-0.01,0.67) | 0.06 | 0.16 (-0.2,0.52) | 0.38 | 0.21 (-0.15,0.57) | 0.25 |
|  | Change in age acceleration, per 5 years** | **0.41 (0.01,0.8)** | **0.04** | 0.22 (-0.19,0.64) | 0.29 | 0.27 (-0.14,0.69) | 0.20 |

* The models with change in age acceleration between visit 2 and visit 5 as the predictor were adjusted for age acceleration at visit 2.

** Covariates: Race, gender, partner status income, education, partner status, EGFR, study site, time since diagnosis and an indicator whether the cancer diagnosis occurred between ARIC visit 2 and 5.

*** Additional covariates: BMI at visit 5, smoking, physical activity, and indicators whether individuals had diabetes or CVD.

**Supplemental Table 3** Sensitivity analysis: Associations of proteomic age acceleration and change (between visit 2 and visit 5) in age acceleration with visit 5 physical and mental quality of life and depression scores, additionally adjusted for chronological age at visit 5, Atherosclerosis Risk in Communities Study

| **Outcome** | **Predictor** |  | **Without cancer**  **(N=1,497)** |  | **With cancer (N=866)** |  |
| --- | --- | --- | --- | --- | --- | --- |
|  | | | **Coefficient (95% CI)** | **P** | **Coefficient (95% CI)** | **P** |
| **Physical Health Score** | Age acceleration, per 5 years | Adjusted** | **-2.37 (-3.64,-1.11)** | **0.000** | **-2.48 (-3.97,-0.98)** | **0.001** |
|  |  | Adjusted, more covariates*** | **-2.92 (-4.12,-1.73)** | **<.0001** | **-2.18 (-3.58,-0.78)** | **0.002** |
|  | Change in age acceleration, per 5 years | Adjusted** | **-2.37 (-3.87,-0.88)** | **0.002** | **-2.52 (-4.21,-0.84)** | **0.003** |
|  |  | Adjusted, more covariates*** | **-3.15 (-4.56,-1.74)** | **<.0001** | **-1.91 (-3.49,-0.33)** | **0.02** |
| **Mental Health Score** | Age acceleration, per 5 years | Adjusted** | -0.38 (-1.35,0.59) | 0.44 | -0.66 (-1.84,0.53) | 0.28 |
|  |  | Adjusted, more covariates*** | 0.00 (-1.01,1.02) | 0.99 | -0.65 (-1.85,0.56) | 0.29 |
|  | Change in age acceleration, per 5 years | Adjusted** | -0.16 (-1.33,1.00) | 0.78 | -1.10 (-2.41,0.22) | 0.10 |
|  |  | Adjusted, more covariates*** | 0.16 (-1.05,1.38) | 0.79 | -1.06 (-2.39,0.27) | 0.12 |
| **Depression Score** | Age acceleration, per 5 years | Adjusted** | **0.48 (0.19,0.78)** | **0.001** | 0.22 (-0.13,0.57) | 0.22 |
|  |  | Adjusted, more covariates*** | **0.57 (0.26,0.88)** | **0.0003** | 0.29 (-0.06,0.65) | 0.10 |
|  | Change in age acceleration, per 5 years | Adjusted** | **0.56 (0.21,0.91)** | **0.002** | 0.27 (-0.13,0.66) | 0.19 |
|  |  | Adjusted, more covariates*** | **0.66 (0.30,1.02)** | **0.0004** | 0.33 (-0.08,0.73) | 0.11 |

** Covariates: Race, gender, partner status income, education, partner status, EGFR, study site; and among those with cancer: time since diagnosis and an indicator whether the cancer diagnosis occurred between ARIC visit 2 and visit 5, chronological age at visit 5.

*** Additional covariates: BMI at visit 5, smoking, physical activity, and indicators whether individuals had diabetes or CVD.

**Supplemental Table** 4 Sensitivity analysis: Associations of proteomic age acceleration and change (between visit 2 and visit 5) in age acceleration with visit 5 physical and mental quality of life and depression scores among cancer survivors, without cancer-specific covariates that were not included in the model among those without a cancer history. N=866, Atherosclerosis Risk in Communities Study

| **Outcome** | **Predictor** | **Adjusted, full model*** | |
| --- | --- | --- | --- |
|  |  | **Coefficient**  **(95% CI)** | **P** |
| Physical health score | Age acceleration, per 5 years | **-1.90 (-3.27,-0.53)** | **0.01** |
|  | Change in age acceleration, per 5 years** | **-1.66 (-3.21,-0.1)** | **0.04** |
| Mental health score | Age acceleration, per 5 years | -0.39 (-1.56,0.78) | 0.52 |
|  | Change in age acceleration, per 5 years** | -0.87 (-2.18,0.44) | 0.19 |
| Depression score | Age acceleration, per 5 years | 0.24 (-0.11,0.58) | 0.18 |
|  | Change in age acceleration, per 5 years** | 0.29 (-0.10,0.69) | 0.15 |

* Covariates: Race, gender, partner status income, education, partner status, EGFR, study site.
